# Investigating nanocatalyst-embedding laser-induced carbon nanofibers for non-enzymatic electrochemical sensing of hydrogen peroxide

**DOI:** 10.1007/s00216-023-04640-8

**Published:** 2023-03-18

**Authors:** Christoph Bruckschlegel, Marc Schlosser, Nongnoot Wongkaew

**Affiliations:** 1 Institute of Analytical Chemistry, Chemo- and Biosensors, University of Regensburg, 93053 Regensburg, Germany; 2 Institute of Inorganic Chemistry, University of Regensburg, 93053 Regensburg, Germany

**Keywords:** Hydrogen peroxide detection, Non-enzymatic sensors, Electrochemical detection, Nanocatalysts, Laser-induced carbon nanofibers, Point-of-need devices

## Abstract

In this present study, we explored the catalytic behaviors of the in situ generated metal nanoparticles, i.e., Pt/Ni, embedded in laser-induced carbon nanofibers (LCNFs) and their potential for H_2_O_2_ detection under physiological conditions. Furthermore, we demonstrate current limitations of laser-generated nanocatalyst embedded within LCNFs as electrochemical detectors and possible strategies to overcome the issues. Cyclic voltammetry revealed the distinctive electrocatalytic behaviors of carbon nanofibers embedding Pt and Ni in various ratios. With chronoamperometry at  +0.5 V, it was found that modulation of Pt and Ni content affected only current related to H_2_O_2_ but not other interfering electroactive substances, i.e., ascorbic acid (AA), uric acid (UA), dopamine (DA), and glucose. This implies that the interferences react to the carbon nanofibers regardless of the presence of metal nanocatalysts. Carbon nanofibers loaded only with Pt and without Ni performed best in H_2_O_2_ detection in phosphate-buffered solution with a limit of detection (LOD) of 1.4 µM, a limit of quantification (LOQ) of 5.7 µM, a linear range from 5 to 500 µM, and a sensitivity of 15 µA mM^−1^ cm^−2^. By increasing Pt loading, the interfering signals from UA and DA could be minimized. Furthermore, we found that modification of electrodes with nylon improves the recovery of H_2_O_2_ spiked in diluted and undiluted human serum. The study is paving the way for the efficient utilization of laser-generated nanocatalyst-embedding carbon nanomaterials for non-enzymatic sensors, which ultimately will lead to inexpensive point-of-need devices with favorable analytical performance.

**Graphical abstract:**

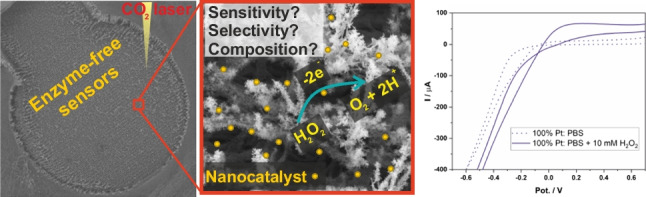

**Supplementary Information:**

The online version contains supplementary material available at 10.1007/s00216-023-04640-8.

## Introduction

Hydrogen peroxide (H_2_O_2_) is one of crucial small molecules present not only in living organisms but also in industries and environment as usage agents or contaminants [1]. Apart from these, H_2_O_2_ is formed in catalytic reactions by many oxidases, e.g., glucose oxidase, which are commonly detected or used in many biosensing platforms [2, 3]. Monitoring H_2_O_2_ both released from cells or inside cellular compartment under physiological conditions is of interest as H_2_O_2_ plays a crucial role in regulating characteristics of cells. Abnormality in H_2_O_2_ level in physiological processes can potentially be an indicator of many diseases, including cancer [4]. Continuous monitoring of H_2_O_2_ through wearable devices has recently received much attention. For example, detecting H_2_O_2_ in wound through a bandage is beneficial for preventing chronic and infectious wounds [5]. Furthermore, H_2_O_2_ in exhaled breath can be a biomarker for asthma, lung cancer, and pulmonary disease [6]. Trends in developing H_2_O_2_ sensors are thus pointed towards onsite and continuous measurement.

Titration [7], spectroscopic [8, 9], fluorescent [10], and chemiluminescent [11] methods have been traditionally employed for detecting H_2_O_2_. Nevertheless, the aforementioned techniques are not suitable for onsite monitoring. Instead, with well-established miniaturized readers, electrochemical methods are more attractive and can realize simpler, faster, more sensitive, and cost-effective measurements. Electrochemical detection of H_2_O_2_ using peroxidase, e.g., horseradish peroxidase (HRP), has been widely used due to favorable sensitivity and selectivity [12]. Nevertheless, cost and instability of enzyme make enzyme-based biosensors less attractive in practice, in particular in resource-limited areas. Therefore, the development of non-enzymatic electrochemical sensors for H_2_O_2_ detection has thus caught our attention.

Since Tour’s group pioneered the utilization of a CO_2_ laser for patterning carbon nanomaterials on commercial plastics, e.g., polyimide (PI) film, a great number of follow-up research has been carried out, which also included the development of smart sensors [13, 14]. Large production capacity, high flexibility in electrode design, cost-effectiveness in terms of materials and instrumentation, and desirable analytical performance of the laser-generated carbon nanoporous materials make the strategy highly attractive to be used in electrochemical sensors, especially towards affordable point-of-need devices. Furthermore, introducing functional entities into polymeric carbon precursors prior to laser pyrolysis allows one to create carbon nanomaterial hybrids, which not only further enhance electron transfer but also enable additional capability, e.g., electrocatalytic reaction that cannot be obtained from pristine carbon nanomaterials.

Previous studies have demonstrated incorporation of metal salts into or onto polymeric carbon precursors and the in situ generation of metal nanocatalysts embedded within the as-obtained carbon nanomaterials [15–18]. For example, Tour’s group prepared PI film containing organic metal salt, e.g., metal complex cobalt(III) acetylacetonate, iron(III) acetylacetonate, or molybdenyl(VI) acetylacetonate, and exposed the film to a CO_2_ laser, enabling the formation of nanocrystal metal oxides embedded within laser-induced graphene [15]. The work opens up for a variety of applications not only in energy-related fields, as shown by the authors, but also in non-enzymatic electrochemical sensors. In our group, instead of preparing a polymer film, we used electrospinning technology to obtain a functional 3D fibrous polymeric carbon precursor. Here, high porosity, immense surface area, and homogeneous dispersion of metal salt realized by electrospinning potentially provide the resulting carbon electrodes with high performance when compared to that of polymeric films. As an example, in our previous study, the electrodes made from laser-induced carbon nanofibers embedded with Ni (Ni-LCNFs) have enabled the detection of glucose in basic medium at sub-micromolar range with negligible interferences [19]. As recently demonstrated also by our group, the 3D porous LCNFs can be further integrated into microfluidic analytical devices, enabling the detection of dopamine in pM range [20].

Various metals have been in fact successfully incorporated to carbon electrodes for H_2_O_2_ detection [21, 22]. For example, Mei et al. proposed a construction of PtNi alloy decorated MWCNTs for non-enzymatic electrochemical sensors of H_2_O_2_ with excellent sensitivity and limit of detection (LOD) in the nanomolar range [23]. Additionally, the PtNi alloy offered the possibility to detect glucose under physiological pH, unlike traditional detection strategies where basic solution medium is required [19]. Recently, Xi et al. reported PtNi nanoparticles with Ni-rich cores and Pt-rich shells that possessed a record high catalytic efficiency with *K*_cat_ of 10^7^ s^–1^, which is much more efficient than pure Pt nanoparticles (a well-known type of efficient peroxidase mimics with similar sizes) [24]. Therefore, the combination of Pt and Ni within LCNFs is of an interest in this study. In particular, we expected to gain new knowledge on fabricating LCNF electrodes with more than a single metal and their applicability for H_2_O_2_ sensing.

Herein, we aim to explore the effect of various combinations of Pt and Ni embedded within LCNFs and reveal their electrochemical behaviors towards non-enzymatic detection of H_2_O_2_. In this study, cyclic voltammetry was used to assess the electrocatalytic behaviors whereas chronoamperometric detection was mainly employed to evaluate the analytical performance. As non-enzymatic electrochemical sensors typically suffer from electroactive interferences, in particular at high applied voltage, we thus thoroughly investigated the effect of each metal composition on selectivity. Furthermore, we investigated some strategies to overcome the signal interferences both in buffer and human serum samples.

## Materials and methods

### Preparation of LCNF

Nanofiber mats were prepared by electrospinning of 15% (w/v) Matrimid® 5218 (Huntsman Advanced Materials BVBA, Belgium) and various ratios and amounts of platin(II)-acetylacetonate ((97%, Sigma-Aldrich, Germany) and nickel(II)-acetylacetonate (95% Sigma-Aldrich, Germany) dissolved in N,N-dimethylacetamide (Merck, Germany) (see supporting information: table S1 and figure S4A). All Ni:Pt ratios are given in mol-percent. The metal salt percentages refer to the dry mass of the polymer. For nanofibers without any metal, a suspension of laser-induced graphene flakes was added to the spinning solution instead of a metal salt. The spinning solutions were stirred at least overnight for homogeneous distribution of all components. The electrospinning was conducted with a rotary drum, tip-to-collector distance of 15 cm, flow rate of spinning solution (10 µL/min), and fiber-deposition substrate (indium tin oxide-coated poly(ethylene terephthalate); ITO/PET, sheet resistivity 60 Ω/sq, 1 ft × 1 ft × 5 mil, Sigma-Aldrich, Germany). For the rotary drum, an ITO/PET piece of 10 cm × 30 cm and two slides of aluminum foil touching both long sides of the ITO/PET were attached to the collector of the rotary drum with adhesive tape to ensure electrical connection between the ITO surface and the grounding. The size of the resulting collecting area was around 9 cm × 25 cm, the applied voltage was 11–12 kV and the drum rotation speed was set to 150 rpm. The optimized spinning time of the rotary drum was 3 h 30 min. The exact temperature and humidity during the spinning are shown in Table S1. The resulting nanofibers were at least dried overnight in the fume hood from organic solvent. The laser-induced carbon nanofibers (LCNFs) were generated by laser scribing the electrospun fibers with a CO_2_ laser (10.6 µm, VLS 2.30, Universal Laser System, Polytech Systeme GmbH, Germany). The laser settings were set to a lasing speed of 60% (1270 mm s^−1^), an image density of 1000 DPI, and a laser power of 1.5 W. If the laser conditions were adjusted (lower laser power or higher lasing speed; see Table S1), the LCNFs were destroyed (electrode burning) with standard conditions. The reasons of such deviations are (i) the various metal compositions with different resulting heat transfer during laser scribing and (ii) low humidity (<40%) during electrospinning which usually results in thinner mats. To keep constant laser conditions in spite of point (i), the total metal salt percentages compared to polymer dry mass were reduced from 25% (LCNFs with 100% Ni) to 15% (LCNFs with 100% Pt).

### Morphology characterization

Elemental mapping and energy-dispersive X-ray (EDX) spectra of LCNFs were investigated with scanning electron microscopy-energy-dispersive X-ray (SEM-EDX) (Zeiss/EVO MA 5 with Bruker XFlash Detector 630 M). The samples were cut with a scissor and not further treated before the measurement.

The morphology of the nanofibers and the various LCNFs was investigated by scanning electron microscopy (SEM, Zeiss/LEO 1530, Germany). The samples were cut with a scissor and platinum-sputtered (1–2-nm layer thickness) before the measurement.

### Electrochemical characterization

The MultiPalmSens4® (PalmSens, Netherlands) with a 3-electrode system (working electrode: LCNF, counter electrode: Pt wire, reference electrode: Ag/AgCl) was used for all electrochemical measurements. The working area of the LCNF had a geometric size of 0.07 cm^2^ and was separated from the contact part of the LCNF with the potentiostat by candle wax. For both measurements, cyclovoltammetry (CV) and chronoamperometry (CA), a drop of the solution (40 µL) was placed on the LCNF while the counter and reference electrodes were reaching into the drop.

CV for effective surface area (ESA) determination was performed from  −0.6 to 1.2 V at 25, 50, 75, 100, 150, and 200 mV s^−1^ in 1 mM ferri/ferrocyanide (in 0.1 M phosphate buffer (phosphate-buffered saline tablet, Sigma-Aldrich, Germany; dissolved in Millipore water; if slight deviations from pH were measured, the solution was adjusted with HCl/NaOH (1 M) to pH = 7.4), 0.1 M KCl).

CVs to see the catalytic effect of the LCNFs were performed from  −0.8 to 0.8 V at 50 mV s^−1^ in phosphate buffer (pH = 7.4) and in 10 mM H_2_O_2_ (Merck, Germany, diluted from a stem solution (CAS-Nr.: 7722-84-1)) in phosphate buffer (pH = 7.4). Firstly, one electrode was measured for 3 cycles in PBS. After that, the same electrode measured a drop of 10 mM H_2_O_2_ in PBS. For the figures, the 3rd cycle of the PBS measurement (usually slight changes of the CV are observed from cycle 1 to cycle 2. From cycle 2 on, the CV is stable) and the 1st cycle of the measurement with H_2_O_2_ were used. In the figures which show CVs, the average CV of n electrodes (see “n” in the figure caption) is shown.

Chronoamperometry (CA) was measured in a non-stirred solution (40 µL placed on the electrode) with a fixed potential of 0.5 V for 100 s. The average signal of the timespan 50–55 s was used as a signal of the CA since this timespan showed low standard deviation between electrodes. As a pretreatment, CAs in pure PBS of one and the same electrode were measured until the signal was constant (usually 10 measurements). After each CA measurement, the drop was removed and a fresh one was placed. After that, a dose-response curve for the analyte (H_2_O_2_) or of a disturbing molecule (ascorbic acid (Merck, Germany), uric acid (Sigma-Aldrich, Germany), dopamine (Sigma-Aldrich, Germany), and glucose (Sigma-Aldrich, Germany)) in PBS was measured with the same electrode. Considering LOD calculation, the last 3 measurements in PBS buffer (the stabilized blank signal) of one electrode were used to calculate the standard deviation of the blank for this electrode. The LOD of a certain LCNF was then calculated by using the average standard deviation of the blank measurement of n electrodes (see “n” in the figure caption) and the average slope of the calibration curve.

To study the impact of sample matrix, CA measurements were initially performed on an electrode 3 times in PBS buffer to establish a stable background current. Then, the buffer was removed. After that, 40 µL of human serum (Sigma-Aldrich, H4522) was dropped (either pure or diluted, i.e., 50% human serum + 50% PBS) onto the same electrode for a 4th CA measurement. Hereby, for every H_2_O_2_ concentration spiked in human serum (0, 50, and 100 µM), a fresh electrode was used. In the case of a polymer coating, 1 µL of the respective polymer solution (5 w% nylon in formic acid) was dropped onto the electrode and evaporated in the fume hood at room temperature for approximately 1 h.

## Results and discussion

### Characterization of nanocatalyst-embedding LCNFs

**Fig. 1:**
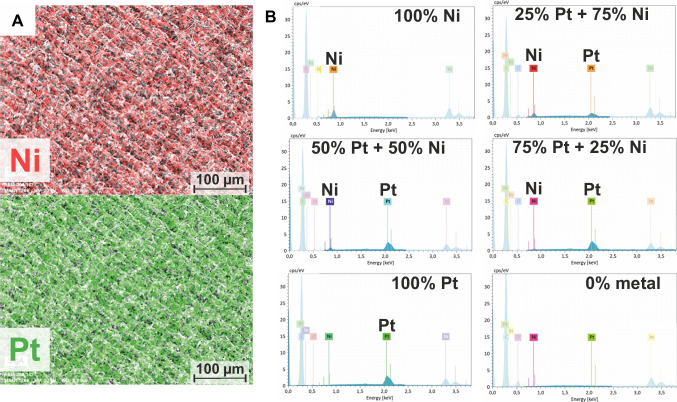
**A** Elemental mapping of Ni and Pt for a (50% Pt + 50% Ni)-LCNFs by SEM–EDX. **B** EDX spectra of various LCNF hybrids focused on Ni and Pt for an area with the size of the SEM image shown in (A), i.e., of around 520 µm $\times$ 350 µm

In a previous study, we found that electrospinning enabled the uniform distributions of Ni throughout the as-spun PI nanofibrous precursor as well as within the LCNF electrodes studied by scanning electron microscopy with energy-dispersive X-ray analysis (SEM-EDX) [19]. Similarly, to ensure no adverse effect resulted from the metal mixture in their distribution, elemental mapping of Ni and Pt atoms was performed for the most representative component, i.e., (50% Pt + 50% Ni)-LCNFs. As shown in Fig. 1A, the EDX signals resulting from Ni and Pt are equally distributed across the whole imaging area. In addition, the intensities of EDX spectra taken from the selected areas for the various LCNF hybrids are highly correlated with the investigated Pt/Ni compositions (Fig. 1B).

An evaluation and a summary of these EDX spectra are given in Table 1. Herein, comparisons between the measured mass concentrations of Pt and Ni atoms presented in the as-prepared LCNFs and their original content in the spinning solution were made. It should be noted that even though inductively coupled plasma mass spectrometry (ICP-MS) is known as a more suitable and accurate technique to determine mass concentration of metals, the challenges encountered during the sample preparation did not allow us to achieve reliable data with ICP-MS. Therefore, the data from SEM-EDX are provided for the discussion.

**Table 1:** Summary of the results from EDX spectra shown in Fig. 1B

Pt content (mol-%)	$\frac{\mathrm m(\mathrm{Pt}(\mathrm{acac}))}{\mathrm m(\mathrm{spinning}\;\mathrm{soln}.)}$ (% wt)	$\frac{\mathrm{m}(\mathrm{Pt})}{\mathrm{m}(\mathrm{LCNFs})}$ (% by EDX)	Ni content (mol-%)	$\frac{\mathrm m(\mathrm{Ni}(\mathrm{acac}))}{\mathrm m(\mathrm{spinning}\;\mathrm{soln}.)}$ (% wt)	$\frac{\mathrm{m}(\mathrm{Ni})}{\mathrm{m}(\mathrm{LCNFs})}$(% by EDX)	$\frac{\mathrm{m}(\mathrm{O})}{\mathrm{m}(\mathrm{LCNFs})}$ (% by EDX)	$\frac{\mathrm{m}(\mathrm{C})}{\mathrm{m}(\mathrm{LCNFs})}$ (% by EDX)
100	6.45	8.5 ± 0.3	0	0	-	2.9 ± 0.6	70 ± 8
75	6.23	8.1 ± 0.3	25	0.69	0.79 ± 0.06	2.8 ± 0.6	70 ± 8
50	5.37	7.2 ± 0.3	50	1.67	2.00 ± 0.09	2.5 ± 0.5	71 ± 8
25	3.5	2.8 ± 0.1	75	2.96	2.5 ± 0.1	5.7 ± 0.9	60 ± 7
0	0	-	100	4.57	5.3 ± 0.2	6 ± 1	71 ± 8

As expected, when increasing Pt or Ni in the spinning dope, the mass of metals in the fibers (rel. to total mass of LCNFs) significantly increased. This implies that during laser scribing, most of the oxygen and nitrogen in acetylacetonate and PI nanofibers (or Matrimid®) was removed [25]. We observed that LCNFs with higher Ni contents, i.e., (25% Pt + 75% Ni) and (100% Ni), contained more oxygen which suggested that during laser scribing, not all PI nanofibers were completely converted into LCNFs, which is also in good agreement with the SEM study of various investigated LCNFs (see Figure S1A, B**)**. This is likely due to the fact that higher thermal conductivity of Ni leads to lower heat localization. In other words, the heat carried by Ni diffuses away faster than that of Pt. The carbon content was nevertheless roughly the same in all investigated LCNFs. In addition to SEM-EDX characterization, electroactive surface area (ESA) was determined for the investigated LCNF hybrids (Figure S2). As well as the peaks of the ferro/ferricyanide redox couple, another anodic and cathodic peak couple appeared at around  +0.7 V and  +0.6 V, respectively, when Ni was present in the LCNFs in relatively high amounts, as reported in our previous study [19]. Such peaks prove the formation of Ni_2_[Fe(CN)_6_], which requires a Ni^2+^ ion, indicating that the nanocatalysts rather consist of NiO than other possible forms, e.g., NiO_2_ and Ni_3_O_4_ [19, 26, 27]. The incorporation of high Pt content in Pt/Ni-LCNF hybrids tended to reduce ESA, which is likely due to greater heat localization behavior of Pt during the lasing process, causing stronger material ablation and less fiber-like structures on top of the usual grid pattern of the laser scriber (see Figure S1A–E). However, when Pt is present alone within LCNFs, a larger amount of Pt is required to increase ESA (see also Table S1), caused by an increased amount of very fine structures arising on top of the usual grid pattern (compare Figure S1E and S1G). This suggests that Pt and Ni mutually facilitated the heat localization during laser carbonization. For LCNFs without any metal, the laser power had to be reduced to 0.9 W in order to avoid the electrode burning, leading to a LCNF without the typical pattern of the laser scriber (see Figure S1F) and a subsequent lower ESA (see Figure S2F). Finally, we have proven that incorporation of metal into PI nanofibers plays a great role in maintaining the integrity of laser carbonization as the LCNFs without metal possessed approx. 2 to 3 times less surface area in comparison to the others (Figure S2F).

### Electrochemical characterization towards H_2_O_2_

**Fig. 2:**
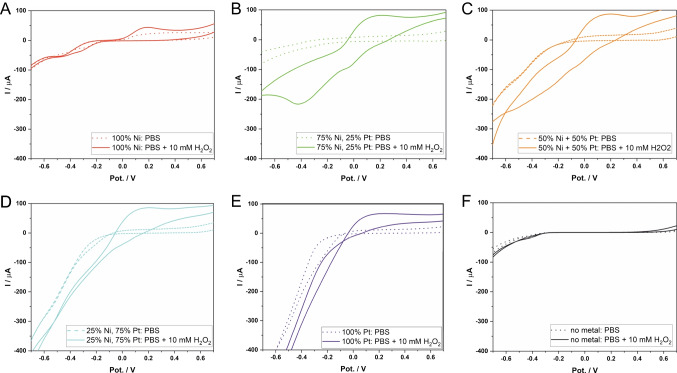
Cyclic voltammograms of LCNFs with various metal compositions where **A** to **E** are from (100% Ni)-LCNFs, (75% Ni + 25% Pt)-LCNFs, (50% Ni + 50% Pt)-LCNFs, (25% Ni + 75% Pt)-LCNFs, and (100% Pt)-LCNFs, respectively (mol-% are given). **F** LCNFs without metal. The characterizations were performed in PBS (pH = 7.4) with and without 10 mM H_2_O_2_ (*n* = 3). The potential (Pot.) is given against Ag/AgCl reference electrode. Scan rate of 50 mV/s was used

Variation of the metal ratio did not only affect the ESA but also played a significant role in the electrocatalytic reaction for non-enzymatic electrochemical detection of H_2_O_2_. Therefore, we systematically characterized the LCNFs loaded with Pt and/or Ni by cyclic voltammetry (CV) to see which LCNF hybrid offered the best catalytic effect towards H_2_O_2_ (Fig. 2). LCNFs containing only Ni nanocatalysts (Fig. 2A) did not show a prominent electrocatalytic activity towards H_2_O_2_ for both oxidative and reductive regions as expected. However, (25% Pt + 75% Ni)-LCNFs (Fig. 2B) were already sufficient to enable the oxidation and reduction of H_2_O_2_, exhibiting oxidation and reduction peaks at around  +0.2 V and  −0.4 V, respectively. The oxidative peak was independent of Pt content when considered within Pt/Ni-LCNF hybrids (Fig. 2B–D). However, the LCNFs containing only Pt exhibited lower anodic peak intensity as well as an undistinguishable cathodic response from the background (Fig. 2E). The decreased anodic peak could be attributed to a smaller ESA comparing to the others (Figure S2E). No significant oxidative or reductive action towards H_2_O_2_ is displayed by the LCNF electrode without any metal (Fig. 2F), which clearly shows that the Pt and/or Ni within LCNF majorly promotes electrocatalytic reaction of H_2_O_2_ while edge/defects of the graphitic sheets play no significant role [28]. Finally, increasing the Pt content resulted in a strong reduction of water, i.e., hydrogen evolution reaction [29].

The results shown in Fig. 2 indicate that Pt plays a major role in the electrocatalytic activity of H_2_O_2_. As suggested by the other studies [30, 31], the oxidation and reduction should start with spontaneous adsorption of H_2_O_2_ (non-electrochemical process) on the free Pt surface:

$$2\mathrm{Pt}+{\mathrm H}_2{\mathrm O}_2\rightarrow2\mathrm{Pt}\left(\mathrm{OH}\right)\left(\mathrm{formula}\;1:\mathrm{adsorption}\right)$$

As Katsounaros et al. showed by quantum chemical ab initio calculations, in an oxidation reaction, another H_2_O_2_ molecule interacts with the two OH groups adsorbed on the Pt surface and is oxidized to oxygen (formula 2: oxidation-1^st^), whereas the resulting water molecules are further oxidized by the Pt surface (formula 3: oxidation-2^nd^) [31], which yields the measurable current at the electrode:

$$2\mathrm{Pt}\left(\mathrm{OH}\right)+{\mathrm H}_2{\mathrm O}_2\rightarrow2\mathrm{Pt}\left({\mathrm H}_2\mathrm O\right)+{\mathrm O}_2\left(\mathrm{formula}\;2:{\mathrm{oxidation}-1}^{\mathrm{st}}\right)$$

$$2\mathrm{Pt}\left({\mathrm H}_2\mathrm O\right)\rightarrow2\mathrm{Pt}\left(\mathrm{OH}\right)+2\mathrm H^++2\mathrm e^-\left(\mathrm{formula}\;3:{\mathrm{oxidation}-2}^{\mathrm{nd}}\;\mathrm{at}\;\mathrm{ca}.+0.2\;\mathrm V\;\mathrm{for}\;\mathrm{Figure}\boldsymbol\;2\right)$$

On the other hand, in a reduction reaction, this OH group will be spontaneously reduced into water, and free Pt sites are available for new coming H_2_O_2_ molecules again [31]:

$$2\mathrm{Pt}\left(\mathrm{OH}\right)+2\mathrm H^++2\mathrm e^-\rightarrow2\mathrm{Pt}\left({\mathrm H}_2\mathrm O\right)\left(\mathrm{formula}\;4:\mathrm{reduction}\right)$$

As reported by Xi et al., a Ni/Pt alloy potentially enhances the catalytic activity towards H_2_O_2_ because of weaker binding of the products to the alloy surface [24]. By density functional theory (DFT) calculations, Xi et al. also showed that the adsorption energy of both the OH species and the O species on the Pt surface decreases by incorporation of Ni [24]. By considering the intermediate steps of formula 4, the O species as follows could appear during reduction reaction:

$$2\mathrm{Pt}\left(\mathrm{OH}\right)\rightarrow\mathrm{Pt}\left({\mathrm H}_2\mathrm O\right)+\mathrm{Pt}\left(\mathrm O\right)\left(\mathrm{formula}\;5:{\mathrm{reduction}-1}^{\mathrm{st}}\right)$$

$$\mathrm{Pt}\left(\mathrm O\right)+2\mathrm H^++2\mathrm e^-\rightarrow\mathrm{Pt}\left({\mathrm H}_2\mathrm O\right)\left(\mathrm{formula}\;6:{\mathrm{reduction}-2}^{\mathrm{nd}}\;\mathrm{at}\;\mathrm{ca}.-0.4\;\mathrm V\;\mathrm{for}\;\mathrm{Figure}\;2\right)$$

As can be seen from Fig. 2B–E, no significant difference was observed for the anodic peaks when introducing Ni into the LCNF hybrids in contrast to the cathodic currents. This is likely due to the fact that Ni tended to reduce the adsorption energy of the intermediates only for the reduction reaction [24], thus modulating catalytic behavior for H_2_O_2_ reduction.

For H_2_O_2_ detection at constant potential (chronoamperometry, CA), one might want to use the (25% Pt + 75% Ni)-LCNF electrode set to  −0.5 V, where a better selectivity towards H_2_O_2_ can be generally realized [32], judging from the cyclic voltammograms in Fig. 2. However, we chose a potential of  +0.5 V instead. This contrary decision can be explained by the fact that, in our experiment, a positive potential usually showed good reproducibility from electrode to electrode and good sensitivity towards H_2_O_2_ when using CA, and finally provided a stable signal during CA measurements. On the contrary, the application of  −0.5 V mostly gave poor reproducibility and signals uncorrelated with H_2_O_2_ concentrations (data not shown). It might be possible that during the oxidative scans of CVs shown in Fig. 2B, the embedded nanocatalyst underwent a necessary oxidation prior to being able to reduce H_2_O_2_. This encourages further intensive studies, which we unfortunately have not been able to address so far in this investigation.

### Influence of various Pt and Ni content on analytical performance

#### Sensitivity and limit of detection

**Fig. 3:**
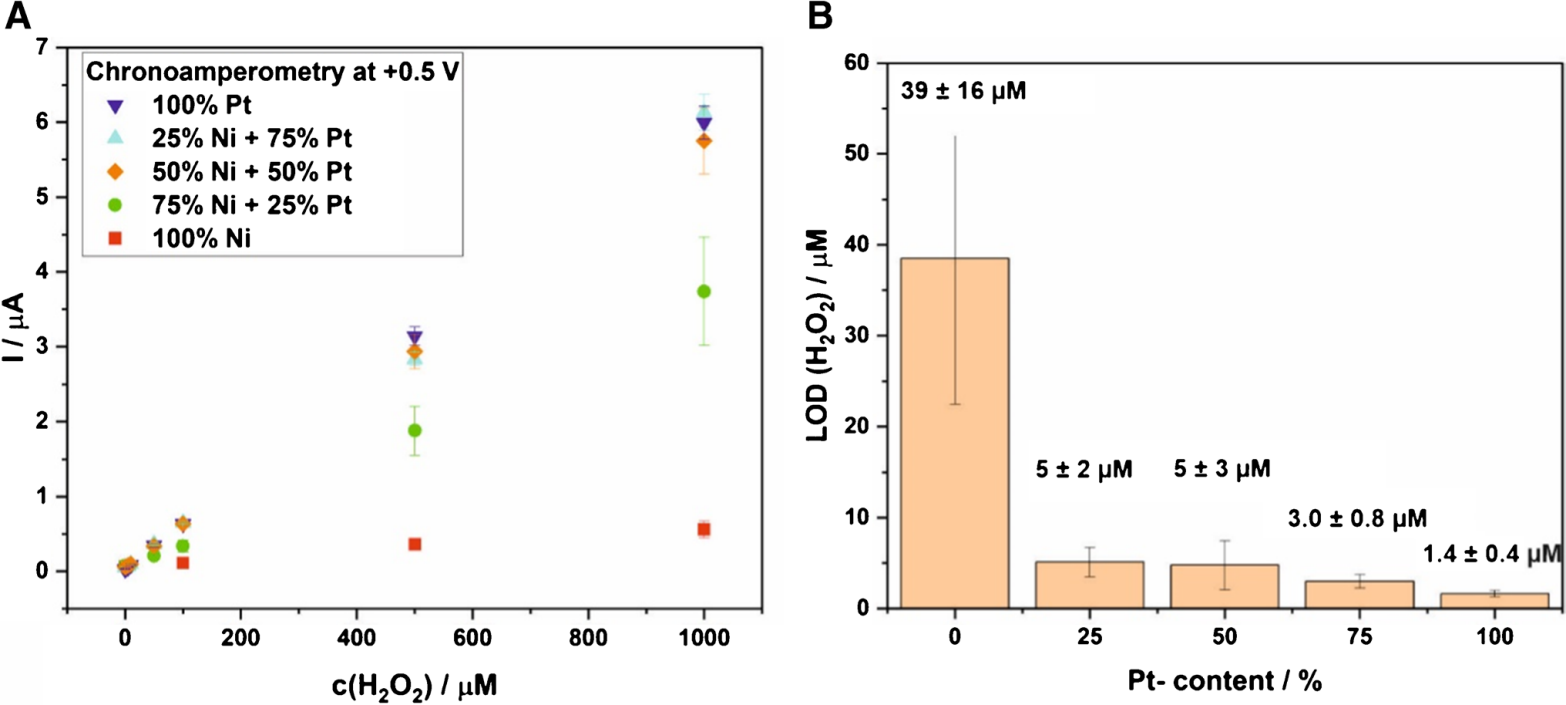
Analytical performance of various LCNFs hybrid electrodes. **A** Calibration curves of the various metal LCNFs for the detection of H_2_O_2_ obtained by non-stirred chronoamperometry at  +0.5 V (*n*≥6). **B** Limit of detection for LCNFs with various Pt ratios

As discussed in the “Materials and methods” section, CA at a positive potential (+0.5 V) was performed for all LCNF electrodes with various Pt/Ni content. Figure 3A shows the average current in the timespan from 50 to 55 s of a chronoamperometric measurement as a function of the H_2_O_2_ concentration in PBS solution (pH = 7.4). The slopes of the resulting calibration curves increased with the Pt to Ni ratio. This was particularly obvious when Pt content rose from 0 to 50% in the Pt/Ni-LCNF hybrids. The sensitivity already reached the maximum at 50% Pt content in the mixture. This indicates that Pt majorly controls the electrocatalytic oxidation of H_2_O_2_. In addition, the result here may suggest that laser scribing probably led to the generation of individual Pt and Ni oxides rather than the formation of a PtNi alloy, as such an alloy was suspected to improve the catalytic reaction of H_2_O_2_, as reported previously [23, 24].

As a next step, we compared the limit of detection (LOD) of the various LCNFs by using the slopes of the calibration curves in Fig. 3A from a concentration range of 5–500 µM H_2_O_2_ (compare with Figure S3), which yielded a good linear correlation. As mentioned earlier in the experimental parts, CA was run for ten times in pure PBS before each calibration to ensure a stable background signal whereas the last three consecutive measurements were used to calculate the standard deviation of the blank within the same electrode (with this standard deviation, a LOQ of 5.7 µM was calculated for the pure Pt electrodes). Since we repeated such measurements also for several different electrodes of the same material type, a measure of uncertainty can be assigned to the standard deviation of the blank measurement. Hence, both the slope and the standard deviation of the blank measurement have an uncertainty, which leads, of course, to a standard deviation of the LOD, calculated by the sum of the partial derivatives multiplied with the respective uncertainty. The LODs of the different LCNFs are shown in Fig. 3B. Although the sensitivities were highly comparable for Pt/Ni-LCNFs with Pt content above 50% in the metal mixture, the LOD tends to decrease, reaching the best value of 1.4 ± 0.4 μM for (100% Pt)-LCNF. This can be explained by the fact that the standard deviation of the blank decreases when Pt content increases, i.e.,  ±5 nA,  ±1.3 nA, and  ±0.7 nA, for (50% Pt + 50% Ni)-LCNFs), (75% Pt + 25% Ni-LCNFs), and (100% Pt)-LCNFs, respectively. This may also imply that addition of Ni causes inhomogeneity of the resultant Pt/Ni-LCNFs hybrids, thus generating higher variation between different electrodes (see also the standard deviations of current response from (75% Ni + 25% Pt)-LCNFs shown in Fig. 3A.

Overall, the analytical performance obtained in this work is highly competitive to the other studies (Table 2), especially considering time, cost, and effort in manufacturing process. The estimated total material cost per electrode with the current electrode design is less than 20 euro cent which is highly suitable for single-use analysis. Furthermore, the high flexibility of material and detection limit in low micromolar range for monitoring H_2_O_2_ without the need of stirring make the nanocatalyst-LCNF hybrids attractive for developing wearable devices.

**Table 2:** Comparison of Pt-LCNF with various Pt- and carbon nanomaterial-based electrodes considering the effort of production, limit of detection (LoD), measurement conditions, linear range, and applied potential

Material	Production steps	LoD (µM) + measurement condition	Linear range (µM)	Pot. vs. Ag/AgCl (V)	Literature
Pt-LCNF	Electrospinning, laser scribing	1.4 ± 0.4 Non-stirred	5–500	+0.5	This study
Pt nanoparticles (NPs)-rGO	rGO^a^, synthesis of Pt-NPs, preparation of Pt-NP-rGO nanocomposite, deposition on glassy carbon electrode (GCE)	0.5 Stirred	2–710	0.0	[33]
Pt-NPs-multi-walled carbon nanotubes (MWCNT)-rGO	MWCNT was bought and pretreated, preparation of freestanding GO^a^-CNT paper, reduction to rGO^a^-CNT paper, Pt sputtering	0.01 Stirred	Up to 25	−0.05	[34]
Pt nanoflower-nitrogen-doped rGO	Synthesis of N-graphene, electrophoretic deposition of N-graphene-modified ITO, electrochemical deposition of Pt nanoflower	0.34 Stirred	1–1000	−0.4	[35]
Pt-NPs-carbon nanofibers (CNF)	Electrospinning, deposition of Pt on NFs, carbonization of NFs, deposition on GCE	1.7 Stirred	5–15,000	−0.2	[36]
Pt-TiO_2_-SWCNT	SWCNT was bought, preparation of SWCNT-film, electrochemical deposition of TiO_2_ structures, photoinduction of platinum nanoparticles	0.73 ± 0.04 stirred	1–1500	+0.7	[37]
Pt_0.5_Au_0.5_ @C-GCE	Preparation of PtAu@C catalyst by microwave-assisted polyol process, catalyst in nafion solution drop-coated on cleaned GCE	2.4 Stirred	7–6500	+0.3	[38]
Pt-MWCNT	Screen-printing carbon black ink onto an Ag-coated PET plastic film, Pt-MWCNT nanohybrid was prepared based on Watanabe method deposition of Pt-MWCNT on screen-printed electrode	Not specified Non-stirred	1000–15,000 100–1000 10–100	+0.3 (vs. screen-printed Ag/AgCl-ink)	[39]
PtNi-MWCNT	MWCNT was bought and acid-pretreated, synthesis of PtNi/C nanocomposites, preparation of catalyst ink and drop-coating on GCE	0.06 Stirred	0.2–24,600	−0.4	[23]

^a^Graphene oxide (GO): was either synthesized or purchased; Reduced graphene oxide (rGO): GO was reduced in a further production step

**Fig. 4:**
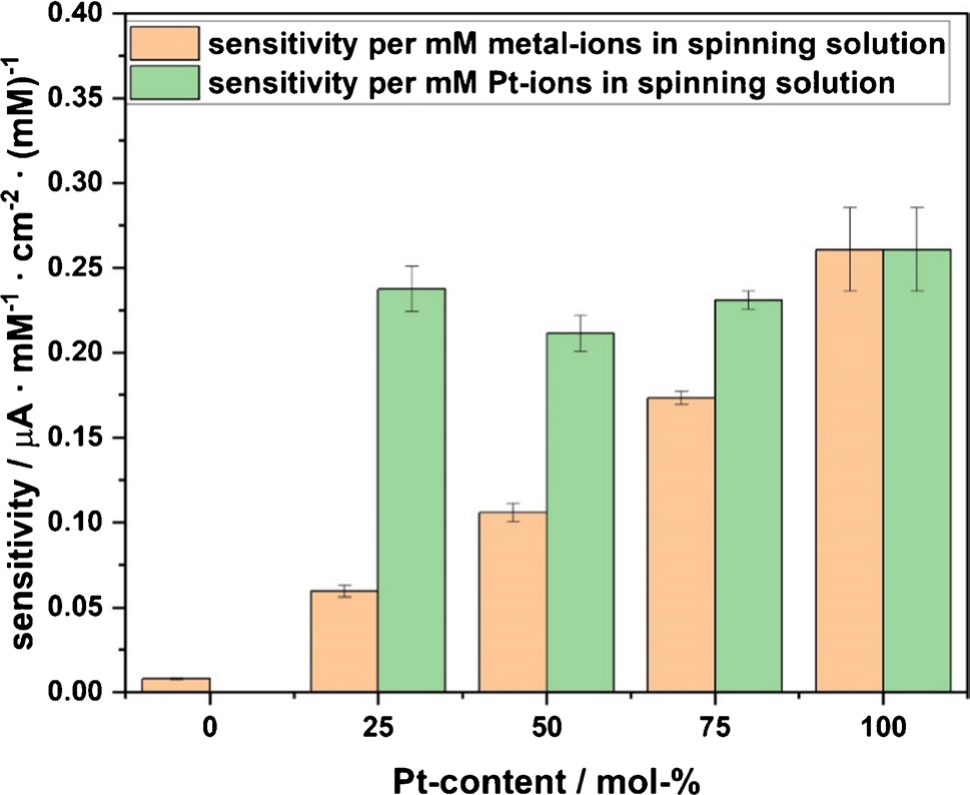
Impact of Pt on the normalized sensitivities shown in Fig. 3A of various Pt/Ni-LCNF hybrids

Since Pt has a higher heat localization capability than Ni, we thus had to reduce the total mass of metal salt added to the spinning solution when increasing Pt content (Figure S4A) to keep the same laser scribing conditions for a better comparison. As a result, we could not obviously see the direct impact of the various metal compositions asserted only from Fig. 3A. In addition, the ESA also slightly varied when LCNFs contained different Pt/Ni combinations (Figure S4B). For a better comparison of the LCNFs, the sensitivities obtained from Fig. 3A were divided by (i) the metal salt or the Pt salt concentration of the respective spinning solution, and (ii) their ESA (Figure S2). As shown in Fig. 4, by increasing the Pt content, the sensitivity per metal salt (concentration of both Ni and Pt ions in the spinning solution) is linearly increased from 0% Pt to 100% Pt whereas the sensitivity per Pt salt (concentration of Pt ions in the spinning solution) is almost constant with the best performance obtained at 100% Pt. These two observations confirmed that increasing the amount of Pt in the LCNFs improved the detection sensitivity of H_2_O_2_ in which Pt is mostly responsible for the sensitivity towards H_2_O_2_, not from bimetallic Pt/Ni alloy as reported by other studies [23, 24]. Instead, adding Ni to the Pt-LCNF tended to poison the Pt nanocatalyst, hindering electrocatalytic reaction of H_2_O_2_.

#### Selectivity

**Fig. 5:**
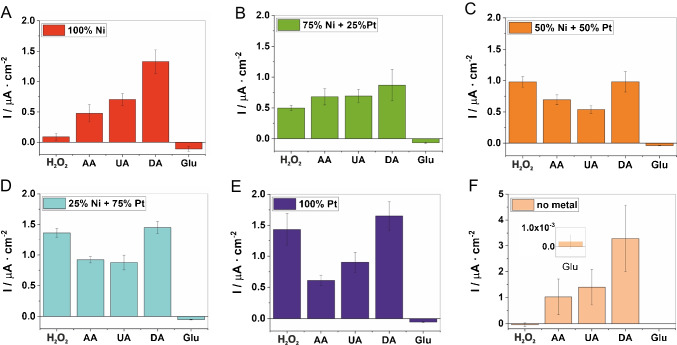
Selectivity of LCNFs with various metal compositions (**A**: 100% Ni, **B**: 75% Ni + 25% Pt, **C**: 50% Ni + 50% Pt, **D**: 25% Ni + 75% Pt, **E**: 100% Pt, **F**: no metal) in PBS solution (pH = 7.4) towards H_2_O_2_, ascorbic acid (AA), uric acid (UA), dopamine (DA), and glucose (Glu) (*n* = 3). The diagrams show the amperometric response of 100 µM substance in PBS from which the signal in pure PBS solution was subtracted

As important as the sensitivity and LOD, the selectivity was studied, especially when applying a high oxidative potential. Therefore, we investigated common electroactive species, which may potentially interfere the detection of H_2_O_2_, e.g., ascorbic acid (AA), uric acid (UA), dopamine (DA), and glucose (Glu). As shown in Fig. 5A–E, the CA signals of 100 µM AA, UA, DA, and Glu in PBS seem to be quite similar for all Pt/Ni-LCNF hybrids whereas current responses from H_2_O_2_ (100 µM in PBS) are highly dependent on Pt content. When using LCNF electrodes without metal (Fig. 5F), the currents from the interferences are prominently seen and approx. 2 times of the signals obtained from LCNFs electrodes which contained metal(s). This is likely due to the fact that edge/defects of the LCNFs are the major cause of poor selectivity where the highly electroactive species, e.g., AA, UA, and DA, can easily oxidize at the sites [40]. These findings emphasize the importance of the exposure of nanocatalyst on the exterior of LCNF surfaces to specifically facilitate electrocatalytic oxidation of H_2_O_2_ [41]. In order to achieve such features, core-shell electrospinning, i.e., PI solution and Pt salt solution are, respectively, fed inside and outside a concentric spinneret, could potentially be applied. Alternatively, spraying or applying Pt salt solution on PI nanofibers prior to laser scribing may efficiently provide nanocatalysts on LCNF surface.

### Investigating strategies for suppressing signals from UA, AA, and DA

In this section, various strategies on suppressing current signals from UA, AA, and DA were mainly investigated as Glu exhibits negligible signal in our current setup. Since the high interfering signals of AA, UA, and DA resulted from the laser-induced graphene itself, one strategy to enhance selectivity is to increase the metal content compared to the dry mass of the PI polymer of the spinning solution. This should principally lead to a higher amount of nanocatalyst present on the LCNF surface while reducing the effect of electrochemical oxidation by the laser-induced graphene. We here continued to investigate the LCNFs containing only Pt as those provided greater analytical performance towards H_2_O_2_ detection than the others.

**Fig. 6:**
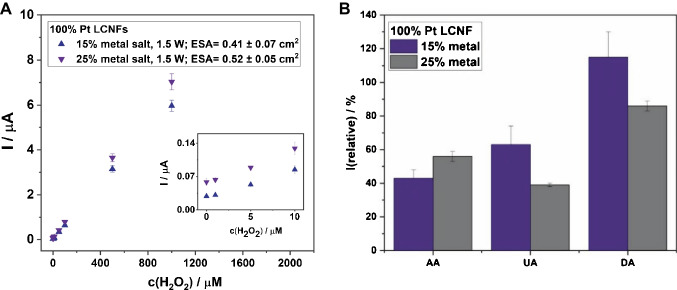
**A** Calibration curves of pure Pt-LCNFs with different metal-to-polymer ratios for the detection of H_2_O_2_ obtained by non-stirred chronoamperometry at +0.5 V (*n* ≥ 3). **B** The % relative signal (at  +0.5 V) of AA, UA, and DA (100 µM in PBS) compared to the signal of a 100 µM H_2_O_2_ solution in PBS for investigated metal ratios (compared to dry mass of the polymer in the spinning solution)

Prior to considering the selectivity, we assessed the effect of Pt content on the detection sensitivity of H_2_O_2_. As shown in Fig. 6A, the increase of Pt content from 15 to 25% slightly improved the detection sensitivity of H_2_O_2_ which is mainly attributed to the elevated ESA. The currents normalized by their ESAs were similar for both cases. However, at an increased Pt content of 25%, the electrodes became more exclusive to UA and DA as indicated by the drop of their signals (relative to that of H_2_O_2_) comparing to 15% Pt (Fig. 6B). The results suggested that UA and DA are more difficult to be oxidized at 25% Pt-LCNFs when compared to H_2_O_2_. It should be noted that even though the interfering signal from DA is relatively high, DA is commonly present in real samples at a very low level, i.e., pico- to nanomolar range [42], and should not cause problems during measurements. To further improve the selectivity against UA, increasing Pt content higher than 25% is promising. In contrast to UA and DA, incorporating more Pt in LCNFs rather facilitated better electron transfer for AA which is known to be an easily oxidizable species [40].

We then further studied different electrode modifications where polymers, i.e., Nafion and nylon, were applied onto the 25% Pt-LCNFs by drop-casting, aiming to suppress the signal interferences from AA. As can be seen in Figure S5, electrodes coated with either Nafion or nylon yielded poorer current ratios of H_2_O_2_ to AA in comparison to unmodified electrodes, which is likely due to the blockage of electroactive surfaces by non-conductive polymers. Nevertheless, we found that using the same amount of polymer, nylon is superior to Nafion which is probably attributed by its non-bulky chemical structure as well as intrinsic highly hydrophilic property that provides less hinderance for electron transfer between analytes and the electrodes.

**Fig. 7:**
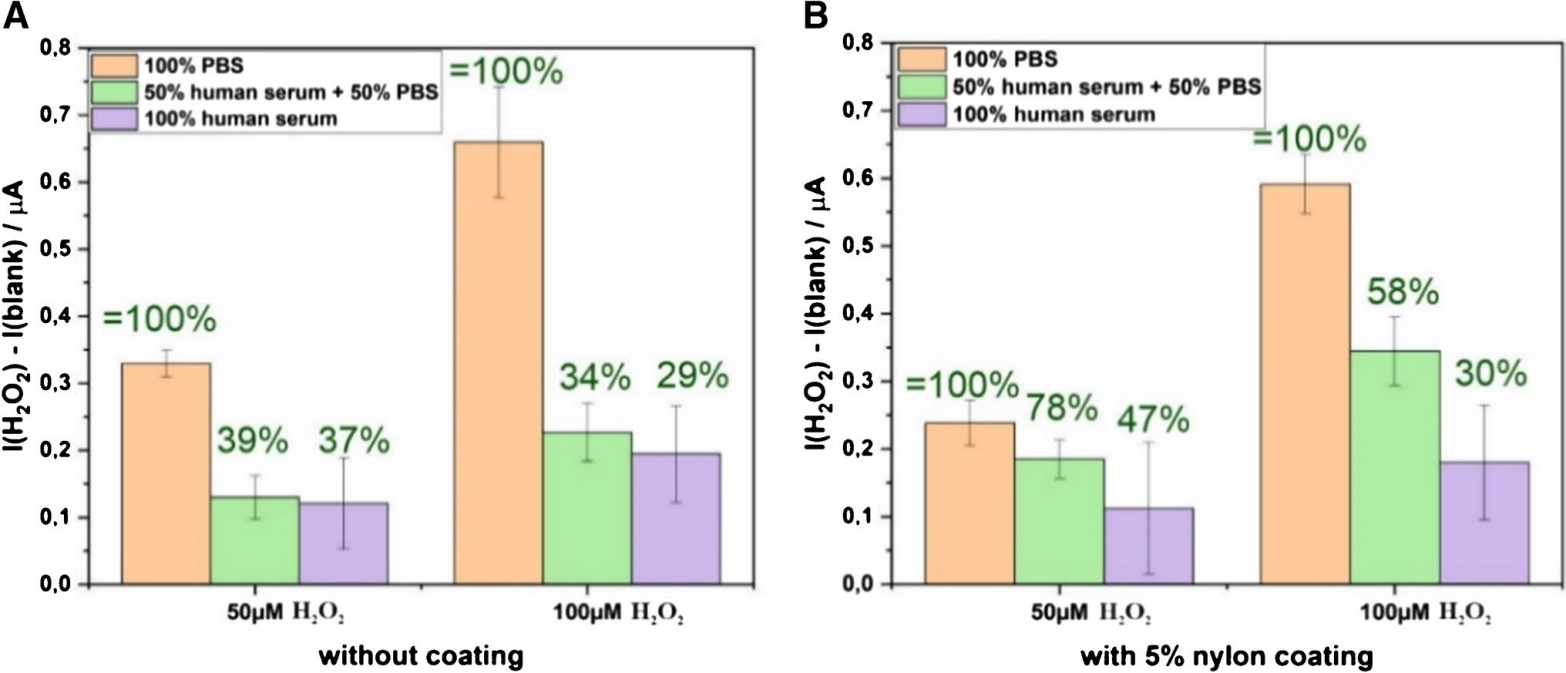
The current response of 50 µM and 100 µM of H_2_O_2_ added to a blank minus the current response of the blank without any H_2_O_2_. Hereby, the blank is either pure PBS, a 50:50 mixture of PBS and human serum, or pure human serum for (**A**) the 25% metal pure Pt-LCNF and for (**B**) the 25% metal pure Pt-LCNF with a 1 µL of 5 w% Nylon coating. The green numbers refer to the recovery, whereas current responses in pure PBS solution are defined as 100% (*n* ≥ 3)

Although nylon did not exhibit a favorable effect on suppressing signal from AA, we found that it improved the recovery of H_2_O_2_ in a matrix of diluted human serum as can be seen from Fig. 7, which has never before been reported [43]. It thus suggests that nylon may be a good candidate as anti-fouling for bioelectronic devices.

## Conclusions

Although the laser-induced carbonization strategy has promised the fabrication of non-enzymatic electrochemical transducers, to the best of our knowledge, there have not been systematic studies which address the effect of nanocatalyst composition on H_2_O_2_ sensing with respect to sensitivity and selectivity when measured under physiological conditions. Here, we demonstrated the incorporation of Pt and/or Ni within electrospun polyimide nanofibers and their conversion into nanocatalysts embedded within laser-induced carbon nanofibers by CO_2_ laser. The carbon nanofiber hybrids were studied towards their capability in non-enzymatic electrochemical detection of H_2_O_2_. Modulation of Pt and Ni content within the nanofiber substrate resulted in distinctive electrocatalytic behavior of H_2_O_2_ oxidation and reduction as well as the analytical performance with respect to sensitivity and selectivity. The combination between Pt and Ni within the carbon nanofibers did not show superior analytical performance as compared to the present of Pt alone. With the optimal hybrid composition, the favorable detection sensitivity and linear range could be attained. However, at the current stage of our development, the hybrid electrodes are not ready for final applications yet as further effort has to be made towards the improvement of selectivity. The results shown in the study suggest further developments on generating electrospun nanofibers where metal salts can be exposed on the exterior to eliminate interfering signal from electroactive species. In addition, to apply other electrochemical detection methods, e.g., differential pulse or square wave voltammetry, may allow distinguishable signals from interfering and H_2_O_2_ species. Furthermore, electrode modification with cationic nanofibers may be considered as anionic interfering species such as ascorbic and uric acid could be electrostatically trapped to the fibers. These findings will ultimately strengthen research and applications of the laser-generated nanocatalysts for non-enzymatic electrochemical sensors.

## Supplementary Information

Below is the link to the electronic supplementary material.

Supplementary file1 (DOCX 1876 KB)
