# Supplementary material for: Investigating nanocatalyst-embedding laser-induced carbon nanofibers for non-enzymatic electrochemical sensing of hydrogen peroxide
Source: Anal Bioanal Chem. 2023 Mar 18;415(18):4487–99. doi: 10.1007/s00216-023-04640-8 (PMC10329077; doi:10.1007/s00216-023-04640-8)
Supplement: Supplementary file 1 — Supplementary file1 (DOCX 1876 KB) [file 216_2023_4640_MOESM1_ESM.docx]

**SUPPORTING INFORMATION**

**Investigating nanocatalyst-embedding laser-induced carbon nanofibers for non-enzymatic electrochemical sensing of hydrogen peroxide**

Christoph Bruckschlegel,*^a^* Marc Schlosser,*^b^* Nongnoot Wongkaew*^a*^*

*^a^* Institute of Analytical Chemistry, Chemo- and Biosensors, University of Regensburg, 93053 Regensburg, Germany

*^b^* Institute of Inorganic Chemistry, University of Regensburg, 93053 Regensburg, Germany

^*^Corresponding author

E-mail: [nongnoot.wongkaew@ur.de](mailto:nongnoot.wongkaew@ur.de)

ORCID: 0000-0002-6118-6182

1. **Optimized conditions for preparing LCNFs-embedded with nanocatalysts at various metal compositions**

**Table S1** Composition of metal precursors, electrospinning conditions, laser settings, and their electroactive surface area determined by Randles–Ševčík equation.

| **Name of metal composition** | **Metal salt compared to dry mass of Matrimid / %** | **Spinning** | **Humidity / %** | **Temp.**  **/ ˚C** | **Laser scriber**  **(power; scribing rate)** | **ESA / cm²** |
| --- | --- | --- | --- | --- | --- | --- |
| 100% Ni | 25 | Rotary drum | 59 | 23 | 1.5W; 60% | 0.52±0.03 |
| 100% Ni | 25 | Rotary drum | 37 | 22 | 1.5W; 60% | 0.54 $\pm$ 0.04 |
| 75% Ni + 25% Pt | 22.5 | Rotary drum | 42 | 23 | 1.2W ; 60% | 0.53±0.03 |
| 50% Ni + 50% Pt | 20 | Rotary drum | 48 | 22 | 1.5W; 60% | 0.59±0.03 |
| 25% Ni + 75% Pt | 17.5 | Rotary drum | 33 | 22 | 1.2W; 75% | 0.39 $\pm$ 0.04 |
| 25% Ni + 75% Pt | 17.5 | Rotary drum | 43 | 22 | 1.5W; 60% | 0.44±0.01 |
| 100% Pt | 15 | Rotary drum | 49 | 26 | 1.5W; 60% | 0.41 $\pm$ 0.06 |
| 100% Pt | 15 | Rotary drum | 54 | 23 | 1.5W; 60% | 0.42±0.04 |
| 100% Pt | 25 | Rotary drum | 48 | 22 | 1.5 W; 60% | 0.52 $\pm$ 0.05 |
| 100% Pt | 25 | Rotary drum | 50 $\pm$ 5 | 22 | 1.5 W; 60% | 0.64 $\pm$ 0.04 |

1. **Morphological characterization of LCNFs by SEM**


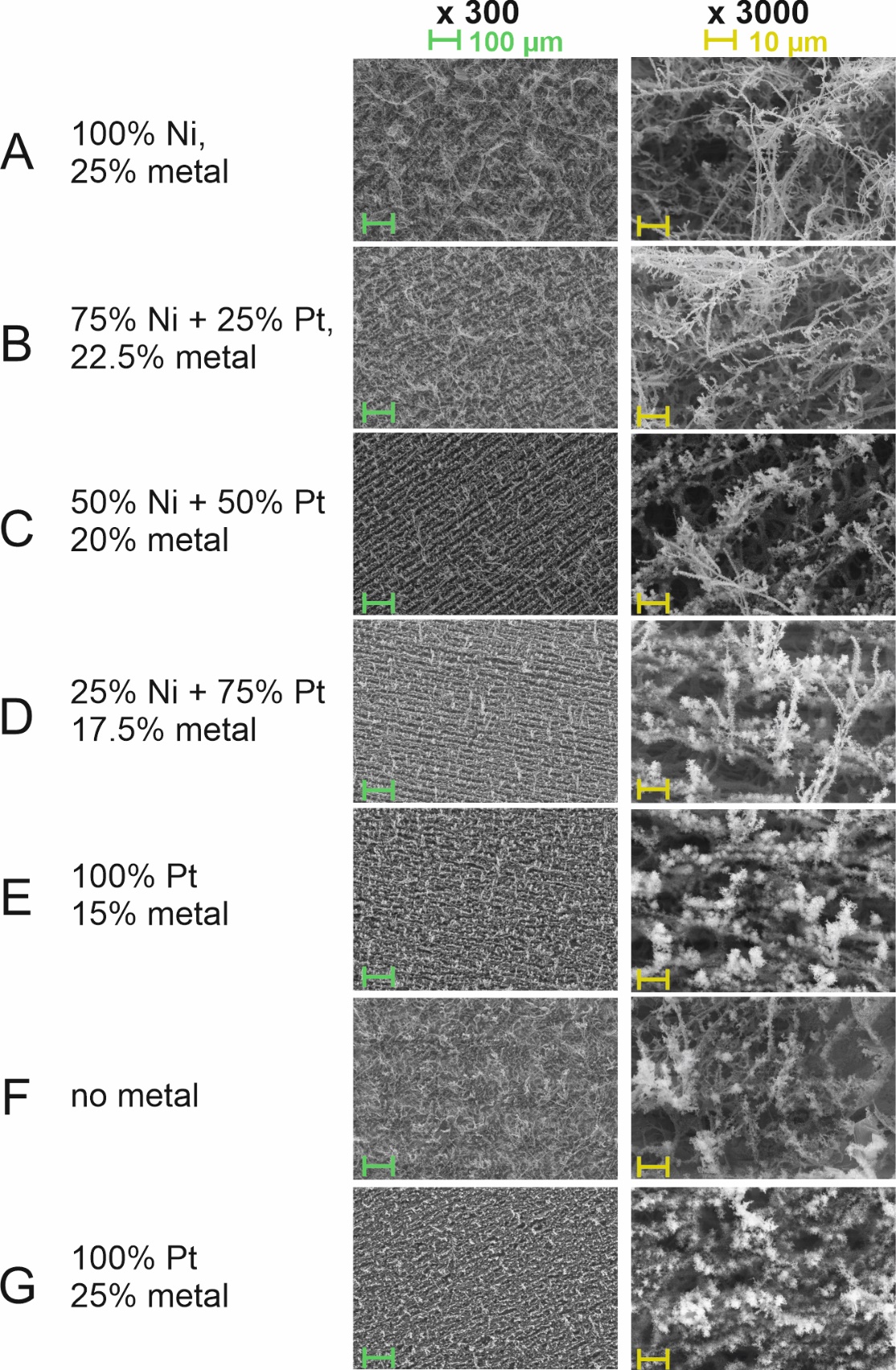


**Figure S1** SEM pictures of various LCNFs with a magnification of 300 in the left and 3000 in the right column. The red arrows point at the fibrous samples that may be under carbonized.

1. **Determining electroactive surface area**


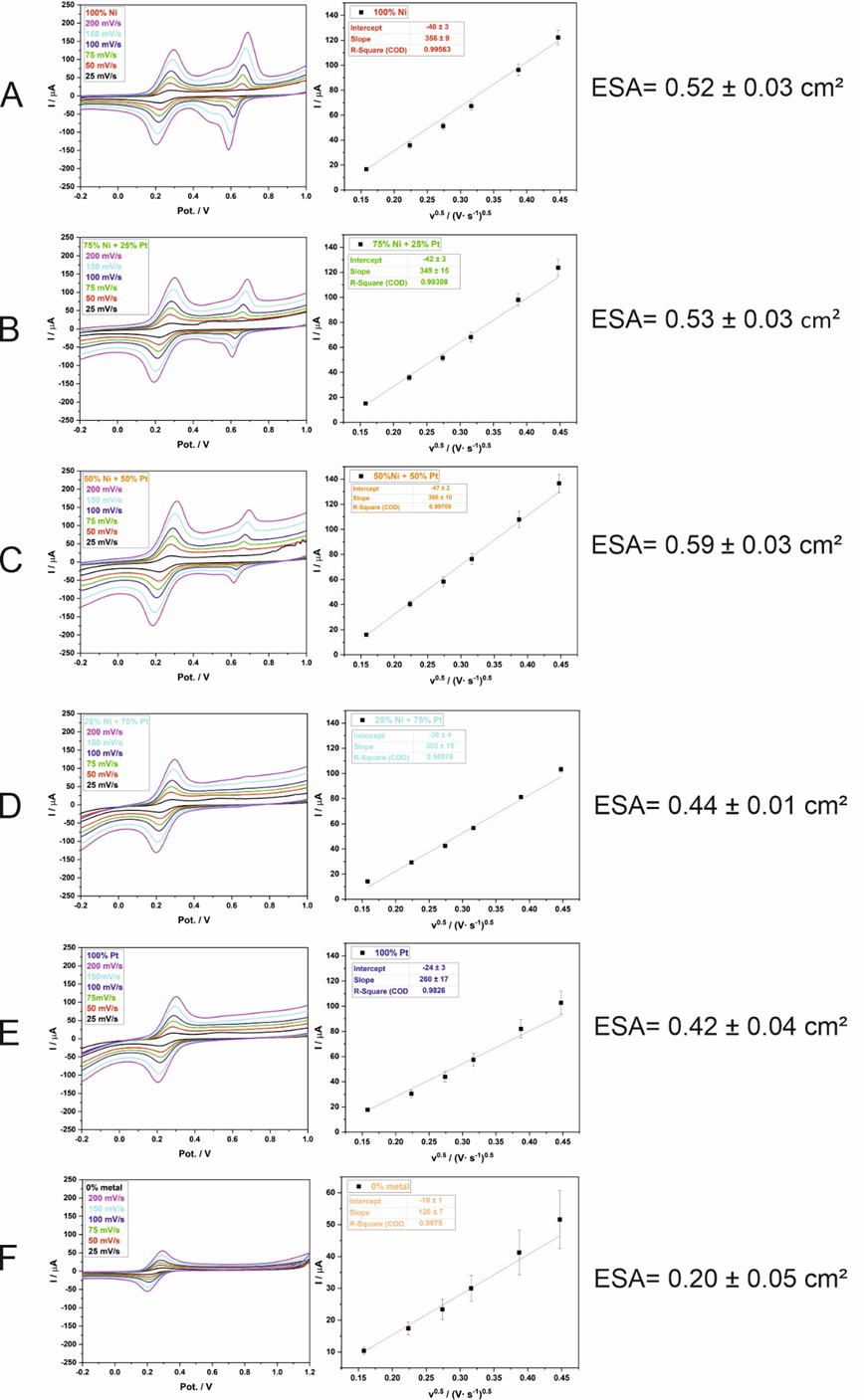


**Figure S2** Cyclic voltammograms at various scan rates of LCNFs (A: 100% Ni, B: 75% Ni + 25% Pt, C: 50% Ni + 50% Pt, D: 25% Ni + 75% Pt, E: 100% Pt, F: no metal) in 1mM ferri/ferrocyanide in PBS-solution (pH = 7.4) (n$\geq$5), the respective Randles-Sevcik plot of the ferri/ferrocyanide peak ($\sim$300 mV, oxidation scan (IUPAC)) and the resulting effective surface area (ESA). The peak couple above +0.6 V in A-C (also slightly visible in D) relate to the formation of nickel hexacyanoferrate species.

1. **The linear range of 100% Pt LCNF**


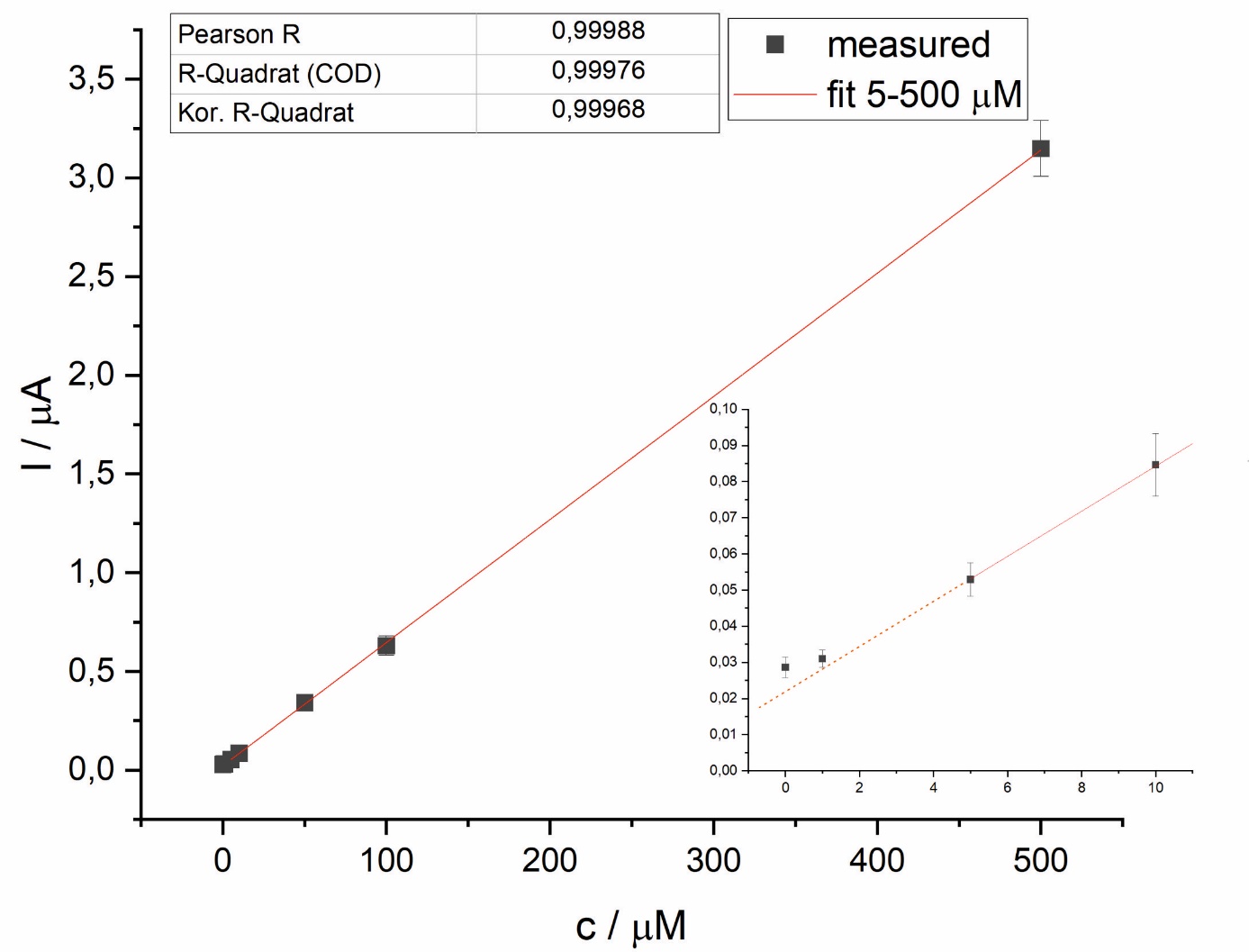


**Figure S3:** LCNFs with 100% Pt data from figure 3A; a plot of low concentrations shows that there is a perfect linear correlation from at least 5-500µM.

1. **Evaluating impact of Pt at various mol-%**


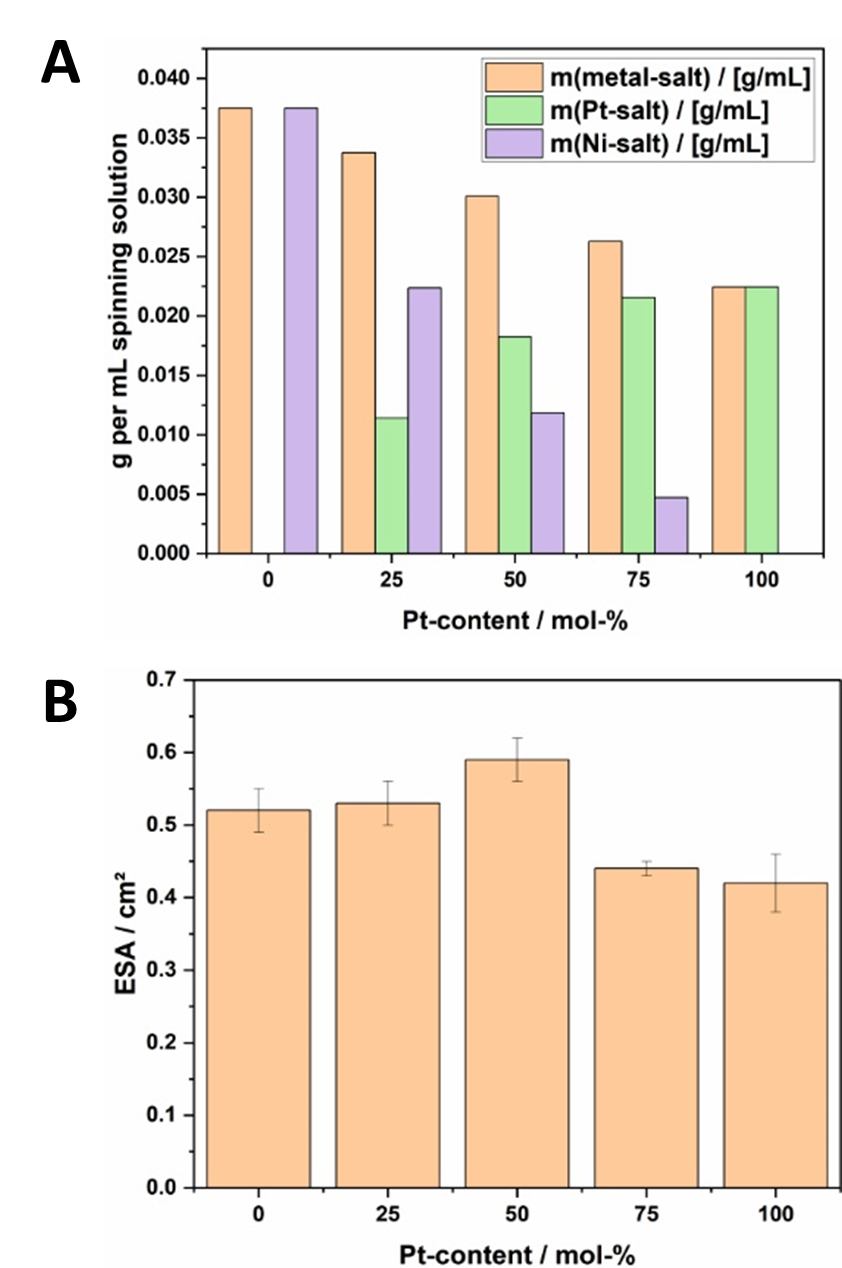


**Figure S4** Mass of the Pt- and Ni-salt per mL spinning solution of the investigated LCNFs and the resulting ESAs from **Figure S1**.

1. **Effect of anionic polymer coating on minimizing signal interference from ascorbic acid (AA)**


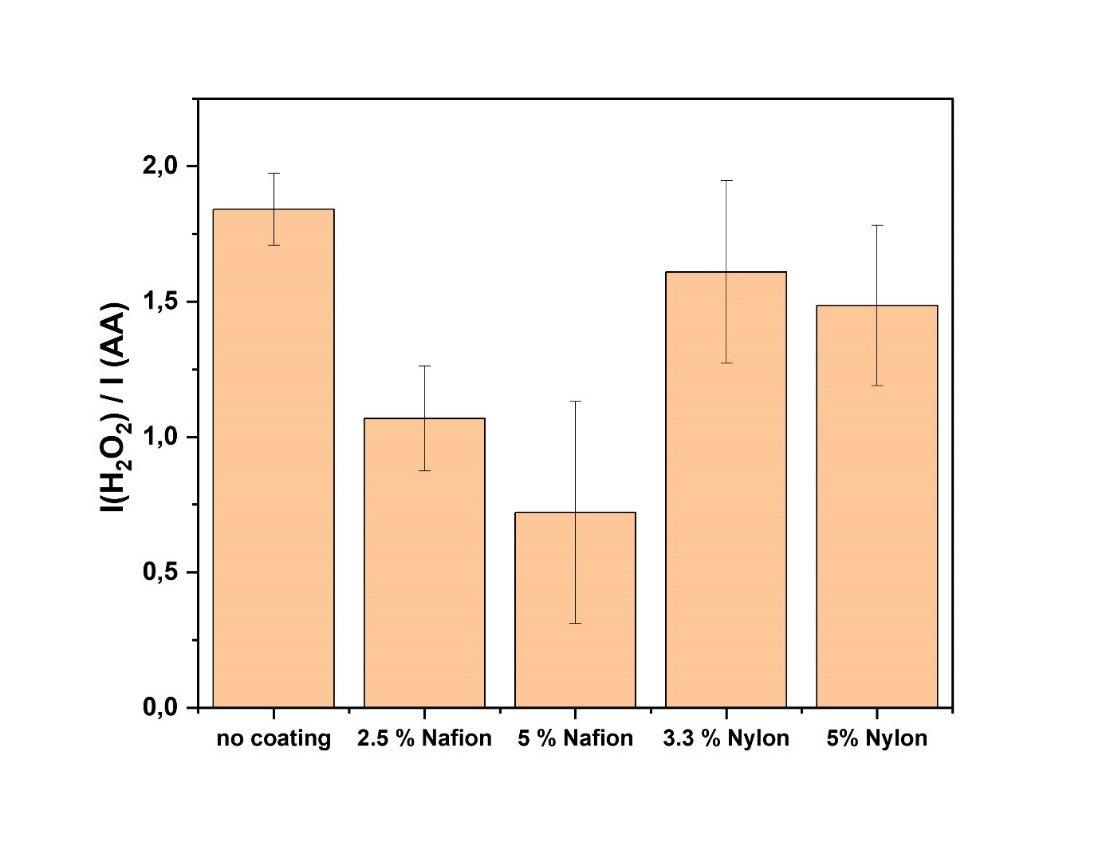


**Figure S5** Effect of polymer coatings of 25% Pt-LCNF on the selectivity shown by the signal ratios of H_2_O_2_ to ascorbic acid (AA). The H_2_O_2_ and AA concentrations were prepared at 100 µM in PBS (n=3).
